# 

**Published:** 1875-11

**Authors:** 


TRANSACTION OF THE MEDICAL SOCIETY OF THE DISTRICT OF COLUMBIA, JULY AND OCTOBER, 1875. Pamphlet; pp. 68.

---

WHAT COURSE SHOULD BE PERSUED WITH AN EYE LOST THROUGH ACCIDENT. By Julian J. Chisolm, M. D. Pamphlet; pp. 8.

---

TWO CASES OF EXOPHTHALMIC GOITRE, ASSOCIATED WITH CHRONIC URTICARIA, ILLUSTRATION OF THE RELATIONS OF THE NERVOUS SYSTEM TO DISEASES OF THE SKIN. By L. Duncan Bulkley A.M., M.D. Pamphlet; pp. 10.

---

THE RELATIONS OF THE URINE TO DISEASES OF THE SKIN. By L. Duncan Bulkley, A.M., M.D. Pamphlet; pp. 33.

---

AMERICAN PUBLIC HEALTH ASSOCIATION. Annual meeting, 1875. President, J. M. Toner, M.D., Washington, D. C.

